# Elevated DOCK4 expression correlates with favorable prognosis and immune infiltration in clear cell renal cell carcinoma

**DOI:** 10.1080/07853890.2026.2642533

**Published:** 2026-03-13

**Authors:** Da-Long He, Fang-Fang Zhu, Jia Wang, Su-Wei Zhu, Shao-Shuai Hou

**Affiliations:** ^a^ College of Basic Medicine, Jining Medical University, Jining, Shandong, China; ^b^ Department of General Practice, Shandong Provincial Hospital Affiliated to Shandong First Medical University, Jinan, Shandong, China; ^c^ The School of Clinical Medicine, Jining Medical University (Affiliated Hospital), Jining, Shandong, China; ^d^ Department of Critical-Care Medicine, Shandong Provincial Hospital Affiliated to Shandong First Medical University, Jinan, Shandong, China; ^e^ Department of Pharmacy, Tengzhou Central People’s Hospital, Affiliated Hospital of Jining Medical University, Tengzhou, Shandong, China

**Keywords:** Clear cell renal cell carcinoma, DOCK4, biomarker, immune infiltration, prognosis

## Abstract

**Background:**

Clear-cell renal cell carcinoma (ccRCC), the most prevalent kidney cancer, has limited treatment options. This study investigated the expression and clinical significance of DOCK4 in ccRCC, hypothesizing that DOCK4 could serve as a prognostic biomarker and correlate with the tumor immune microenvironment (TIME) and immunotherapy response.

**Methods:**

We comprehensively analyzed data on DOCK4 expression levels and prognostic outcomes in ccRCC patients derived from tissue microarray (75 pairs) and the dataset (532 cases). Based on univariate and multivariate Cox regression analyses, we constructed a prognostic nomogram and validated its predictive accuracy and risk stratification ability using calibration curves and Kaplan‑Meier survival analysis. Immune cell infiltration analysis was conducted to investigate DOCK4’s role in the TIME.

**Results:**

Analysis of DOCK4 expression in ccRCC tissues revealed its notable overexpression. High DOCK4 expression was associated with more favorable pathologic staging and improved survival outcomes. DOCK4 shows high discriminatory power between tumor and normal samples, with an area under the receiver operating characteristic (ROC) curve (AUC) of 0.819. This association was confirmed using tissue microarray analysis. Further investigation indicated that high DOCK4 expression may play a crucial role in modulating the TIME, potentially enhancing susceptibility to immunotherapy and strengthening immune surveillance mechanisms.

**Conclusions:**

DOCK4 is a promising prognostic biomarker in ccRCC, with high expression indicating favorable pathologic staging and improved survival. Its role in modulating the TIME suggests that DOCK4 could also serve as a therapeutic target, potentially guiding future immunotherapy strategies for this aggressive malignancy.

## Graphical Abstract

**Figure:**
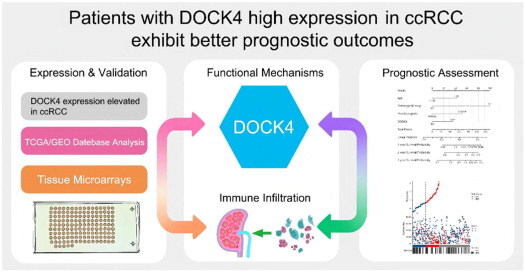

This study integrates microarray and multiple database results to demonstrate that DOCK4 serves as a prognostic biomarker for clear cell renal cell carcinoma (ccRCC) and suggests its potential as a basis for guiding immunotherapy sensitivity assessment.

## 1. Introduction

Renal cell carcinoma (RCC) is the most prevalent kidney cancer type [1], which clear cell renal cell carcinoma (ccRCC) is the most prevalent subtype of kidney cancer, representing a significant clinical challenge, and it has emerged as a major global health concern [2]. The Global Cancer Statistics (2020) estimated that approximately 430,000 new cases of RCC were diagnosed that year [3]. ccRCC is more prevalent in men than in women, and the median time to diagnosis is about 60 years old [4]. Many patients with ccRCC often remain asymptomatic or experience only nonspecific symptoms until the disease reaches an advanced stage [5]. Therefore, identifying reliable and effective metrics to assess the prognostic outcomes of ccRCC patients is crucial for optimizing clinical management. Such metrics are essential for tailoring personalized treatment strategies, improving patient prognosis, and facilitating early interventions that could significantly enhance survival rates and quality of life.

Dedicator of cytokinesis protein 4 (DOCK4), is a member of the CDM gene family and encodes a regulator of small GTPases that promotes the conversion of GDP to active GTP [6,7]. DOCK4 possesses a distinctive N-terminal SH3 structural domain and a C-terminal proline-rich region, with the latter exhibiting structural variations unique to each member of the DOCK family, highlighting the diversity and specificity of its functional roles [6]. This structural complexity may contribute to its unique mechanisms of action and potential involvement in various cellular processes.

DOCK4 is involved in regulating intercellular adhesion and plays a crucial role in cancer cell migration [8]. Nevertheless, the study of DOCK4 for tumor migration varies considerably across cancers. Inactivation of DOCK4 in mouse osteosarcoma cells results in impaired adhesion junction formation [6]. Moreover, DOCK4 is a key factor in breast cancer cell migration by activating downstream target proteins [8,9]. However, it has been proposed that reduced expression of DOCK4 could serve as a potential marker for poor prognosis in renal cancer, although comprehensive and specific analyses to fully understand its clinical significance are currently lacking [10]. This suggests that DOCK4 expression levels could serve as a potential biomarker for the prognostic stratification of ccRCC patients. Despite its potential significance, DOCK4 has yet to be extensively and systematically studied or reported in the ccRCC. Further investigation into the relationship between DOCK4 expression and patient outcomes is essential to validate its role as a prognostic indicator and to explore its potential as a therapeutic target.

In this study, we comprehensively examine the prognostic significance and potential immunomodulatory role of DOCK4 in ccRCC. Analyses of The Cancer Genome Atlas (TCGA) and Gene Expression Omnibus (GEO) databases revealed a significant upregulation of DOCK4 in ccRCC tissues. To further explore the molecular mechanisms underlying this observation, we employed Gene Ontology (GO) and Kyoto Encyclopedia of Genes and Genomes (KEGG) pathway enrichment analyses, as well as Protein-Protein Interaction (PPI) network analysis, to identify key genes and signaling pathways associated with DOCK4. A prognostic nomogram was constructed and validated using Cox regression, calibration, and Kaplan‑Meier survival analysis, which revealed that high DOCK4 expression is closely associated with favorable clinical outcomes. Notably, our investigation also highlighted a strong correlation between DOCK4 expression and immune cell infiltration, suggesting that DOCK4 may play a pivotal role in shaping the tumor immune microenvironment. Then, patients with elevated DOCK4 expression demonstrate enhanced responsiveness to immunotherapy, indicating its potential as a predictive biomarker for therapeutic outcomes in ccRCC. In summary, DOCK4 serves as a critical biomarker for prognostic assessment, offering valuable insights into disease progression and helping to guide personalized treatment approaches for improved patient outcomes.

## 2. Materials and methods

### 2.1. Data collection

The expression profile of DOCK4 in pan-cancer was obtained using the TIMER2.0 database (http://timer.cistrome.org/). Transcriptomic data from 532 cases of ccRCC and 72 normal samples were collected from the TCGA (https://portal.gdc.cancer.gov/) database, covering clinicopathologic and prognostic survival information of the patients [11]. For subsequent analysis, RNA-seq data were converted from FPKM format to TPM (transcripts per million reads) format. DOCK4 expression was validated using microarray data from GSE40435, GSE16449, and GSE53757.

### 2.2. Analysis of DEmRNA

Differentially expressed mRNA (DEmRNA) was obtained using the “Limma” software package [12], with the filtering condition that the absolute value of the change in log2 multiplicity (|logFC|) was >2 and the corrected P-value (P.adj) was <0.05, and by using the R language [13]. The mRNAs co-expressed with the target genes were obtained. In addition, the volcano map of the mRNAs was visualized using the “ggplot2” software package [14], and the heat map of the target genes co-expressed with their mRNAs.

### 2.3. Human ccRCC tissue microarray

The human ccRCC tissue microarrays (Shanghai Outdo Biotech Co., Ltd., Shanghai, China) were constructed from specimens of 75 ccRCC patients who underwent radical resection. The surgical procedures were performed between July 2006 and November 2008, with follow-up conducted until September 2012. All patients had not received adjuvant chemotherapy or radiotherapy. The use of human samples in this research has been conducted with informed consent and approved by the Ethics Committee of Shanghai Outdo Biotech Company Limited (SHYJS-CP-1407019; 2014.07.01-2099.12.31). All participants signed informed consent forms.

### 2.4. Immunohistochemistry

Microarrays were first baked in an oven at 65 °C for 2 h and then deparaffinized using xylene and gradient ethanol. Goat serum was used for sealing, and then the sections were incubated with DOCK4 antibody (1:100 dilution, ProteinTech, Cat No. 21861-1-AP) at 4 °C overnight. After that, the samples were co-incubated with secondary antibody (goat anti-rabbit antibody, Beijing Zhongshan Biotechnology Co., Ltd.) for 1 h at room temperature. Next, the samples were stained with DAB, re-stained with hematoxylin and differentiated with hydrochloric acid and alcohol, respectively. Finally, images were acquired using an Olympus microscope imaging system (Olympus Corporation, Tokyo, Japan).

### 2.5. Immunohistochemistry scoring

Double-blind scoring was performed by two experienced associate senior pathologists according to the following method (Immune Response Scoring, IRS): the intensity of cellular staining was divided into 4 grades: no positive staining (negative) was scored as 0 points, light yellow (weakly positive) was scored as 1 point, brownish-yellow (positive) was scored as 2 points, and brownish (strongly positive) was scored as 3 points. The percentage of positive cells is categorized into 4 grades: ≤25% is scored as 1 point, 26%-50% is scored as 2 points, 51%-75% is scored as 3 points, and >75% is scored as 4 points. The two scores were multiplied to obtain the final score.

### 2.6. Analysis of functional enrichment of DOCK4-related genes using the LinkedOmics database

GO analysis is a bioinformatics tool used to annotate genes and their associated products, covering three components: cellular components (CC), molecular functions (MF), and biological processes (BP) [15]. The KEGG is a collection of databases storing information on genomes, biological pathways, diseases, and chemicals [16]. The LinkedOmics database (http://www.linkedomics.org/login.php) integrates multi-omics data from 32 TCGA cancer types and 10 CPTAC cancer cohorts [17]. We used the “LinkFinder” module of this database to screen differentially expressed genes associated with DOCK4 in the TCGA ccRCC cohort, and the “Function” module to carry out GO and KEGG pathway analysis.

### 2.7. Protein-Protein Interaction network analysis

The PPI network for DOCK4 was constructed using the online database STRING (https://string-db.org/). The top 10 binding proteins interacting with DOCK4 were obtained from the STRING database and GO and KEGG pathway analysis was carried out.

### 2.8. Survival analysis

Kaplan-Meier survival analysis was performed on all clinicopathologic features of ccRCC patients, and *P*-values were calculated by the log-rank test. The patients’ expression data were categorized into DOCK4 low and high expression groups using the curve cut-point function of the survminer package (version 0.4.9) in R. The patients’ expression data were categorized into DOCK4 low and high expression groups. Subsequently, univariate and multivariate Cox regression analyses were used to identify independent prognostic factors [18]. *P* < 0.05 indicates statistical significance.

### 2.9. Construction and validation of DOCK4 differential expression and prognostic models

The differential expression accuracy of DOCK4 in ccRCC was assessed by receiver operating characteristic (ROC) with the “pROC” (version 1.18.0) software package. To assess the prognostic significance of DOCK4 in ccRCC, univariate and multivariate Cox regression analyses were performed using the “survival” (version 3.3.1) and “rms” (version 6.3.0) software packages to assess gender, age, T/N/M stage, pathological grade, tumor location, and other patient characteristics on overall survival (OS). To visualize the Cox regression results, forest plots were drawn using the “ggplot2” software package. Based on the independent risk factors screened in the Cox proportional risk regression analysis, a time-dependent column-line diagram for the prediction of OS was constructed, and the effects of each factor were quantitatively assessed in conjunction with the risk factor map. The goodness of fit of the column chart was assessed by plotting calibration curves. Column plots and calibration curves were generated with the RMS package (version 6.2-0), while survival analysis was performed with the survival package (version 3.2-10) to predict OS [19].

### 2.10. Immune infiltration analysis and immunotherapy sensitivity assessment

To explore the potential association of DOCK4 expression with tumor microenvironment, the immunity score, stroma score, and composite score (ESTIMATE score) of renal clear cell carcinoma samples were calculated based on the TCGA database using the ‘ESTIMATE’ software package [20].

The relative concentration of each type of immune cell was quantitatively graded based on the gene expression profiles of each tumor sample using the GSVA R package (version 1.34.0) [21]. Calculate immune cell infiltration in corresponding cloud-based data using markers from 24 immune cell types [22]. The association between DOCK4 expression and immune cells was explored using Spearman correlation analysis, and the differences in immune infiltration levels between DOCK4 high/low expression groups were compared by Wilcoxon rank sum test.

Detailed analysis of survival parameters under different immune cell enrichment or depletion conditions was performed using the Kaplan-Meier survival analysis tool (https://kmplot.com/analysis/) [23]. Spearman correlation analysis was used to explore the association between DOCK4 expression and immune checkpoint genes (including *CD274*, *CTLA4*, *HAVCR2*, *LAG3*, *PDCD1*, *PDCD1LG2*, *TIGIT*, *SIGLEC15*) in ccRCC. Based on the Cancer ImmunoAtlas (TCIA, http://tcia.at/) [24], predicting the effect of DOCK4 expression-associated immunotherapy.

### 2.11. Statistics

All statistical analyses were done using R software (version 3.6.3) and Graphpad PRISM 8. Statistical significance was analyzed using the Wilcoxon rank sum test and paired-samples t-test. Correlations between clinical characteristics and DOCK4 expression were assessed using the Wilcoxon rank sum test and one-way logistic regression. Prognostic analyses were performed using one-way and multifactorial Cox regression analyses. Immunologic features of DOCK4 in ccRCC were explored by Spearman correlation analysis and Wilcoxon rank sum test. In all analyses, *P*< 0.05 was considered statistically significant.

## 3. Results

### 3.1. DOCK4 expression is up regulated in ccRCC

**Figure 1.**
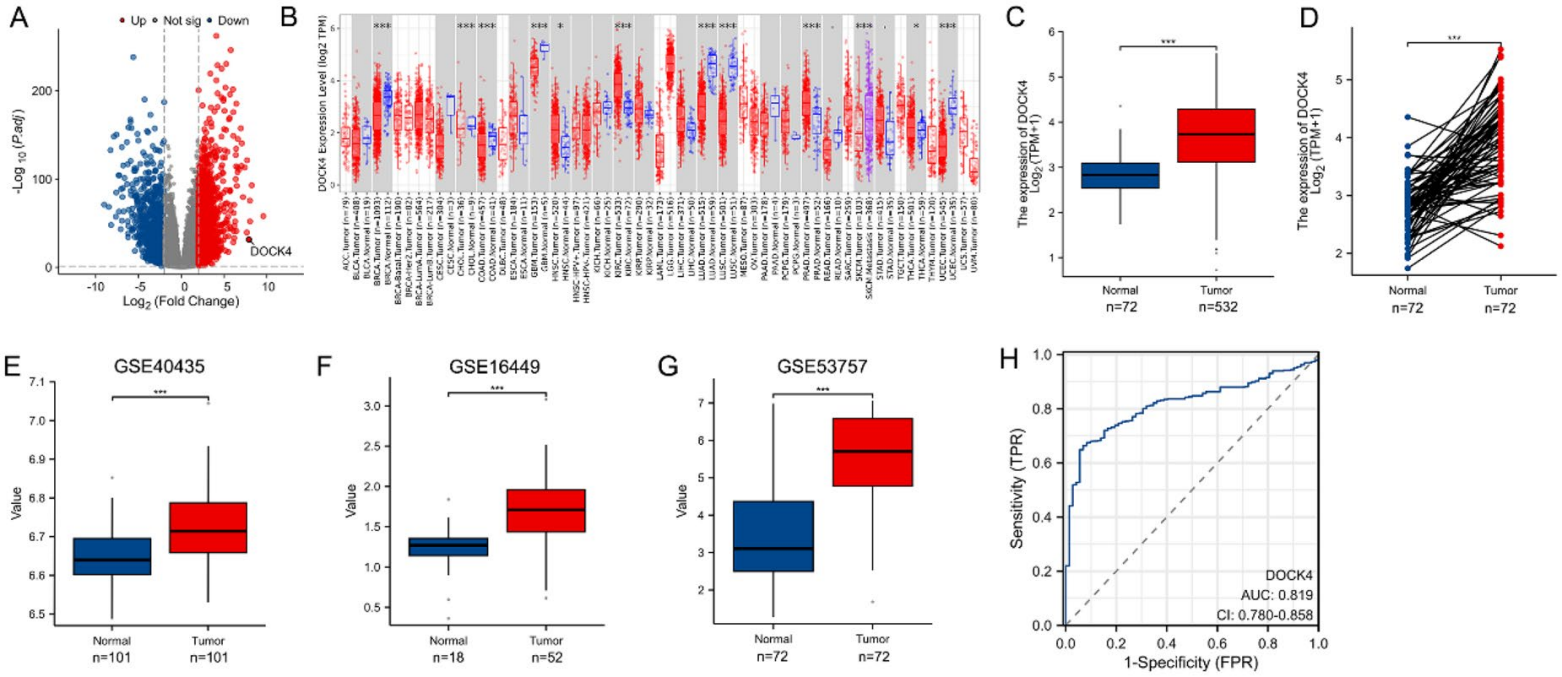
Expression levels of DOCK4 in different tumor typologies and ccRCC. A: mRNA distribution in ccRCC and identification with DEmRNA. Volcano plot of 2367 DEmRNA, red indicates up-regulated mRNA, blue indicates down-regulated mRNA. X-axis is LogFC, Y-axis is - Log10 (*P*.adj). B: Differential expression of DOCK4 in pan-cancer samples from the TIMER2.0 database. C: DOCK4 expression in ccRCC and unpaired normal tissues in the TCGA database. D: ccRCC and paired normal tissues with DOCK4 expression in the TCGA database. E–G: DOCK4 mRNA expression between ccRCC and normal tissues based on GSE40435 (E), GSE16449 (F), and GSE53757 (G) datasets. H: ROC curves of DOCK4 in ccRCC. ROC, Receiver operating characteristic. *P < 0.05,**P < 0.01, ***P < 0.001

We identified 2367 DEmRNAs in ccRCC versus normal kidney tissue samples, of which 1517 were up-regulated and 850 were down-regulated, with the volcano map distribution as shown in Figure 1A. DOCK4 is prominently located on the right side of the volcano plot, indicating that it is upregulated in ccRCC. We screened 33 cancer types by TIMER2.0 to investigate the mRNA expression of DOCK4 (Figure 1B). The results showed that there were significant gene expression differences in 12 of the 33 cancer types, and the expression of DOCK4 tended to decrease in most tumor tissues, except for renal clear cell carcinoma. In this study, we focused on the expression differences of DOCK4 in ccRCC. After reviewing the relevant data in the TCGA database, it was found that the expression of DOCK4 was elevated in ccRCC, and this phenomenon was also confirmed in 72 paired tissues (Figure 1C and D). In addition, the GSE40435, GSE16449, and GSE53757 datasets further validated the high expression of DOCK4 in ccRCC (Figure 1E–G). ROC curve analysis demonstrated that DOCK4 could effectively distinguish ccRCC tissues from normal renal tissues, with an area under the curve (AUC) of 0.819 (Figure 1H), indicating that DOCK4 possesses high diagnostic value for ccRCC.

### 3.2. DOCK4 is upregulated in ccRCC tissue microarrays and has a positive prognosis

**Figure 2.**
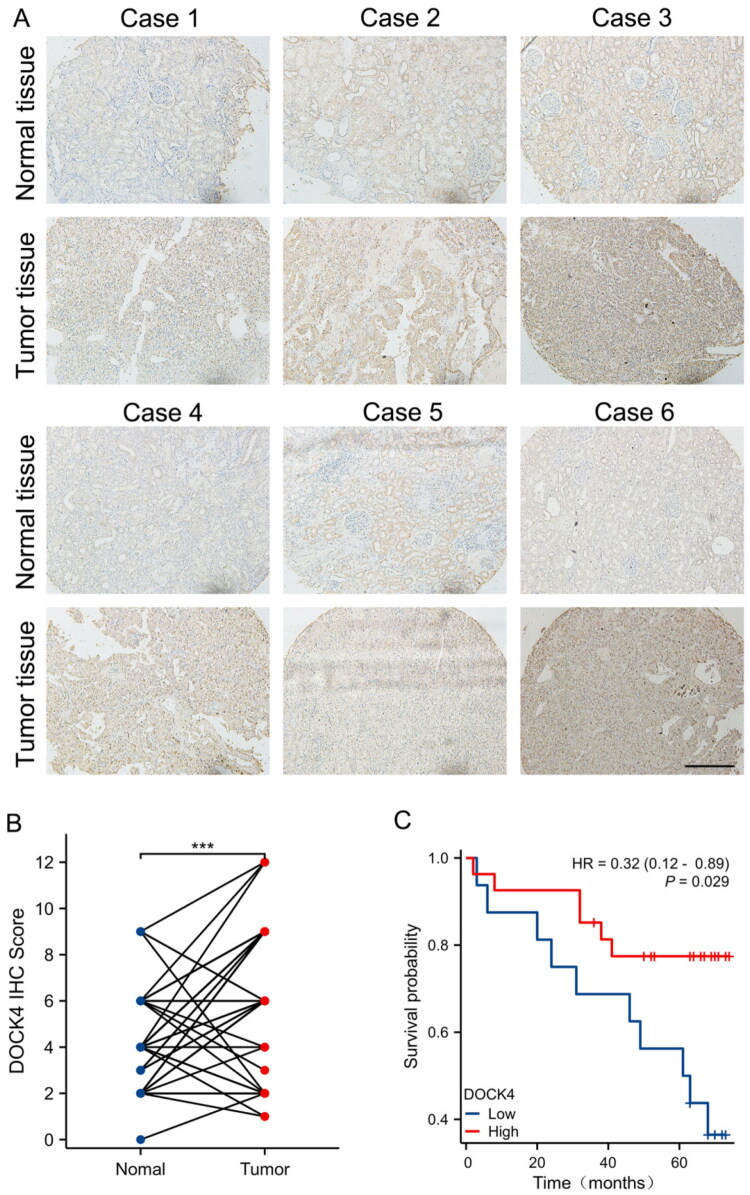
DOCK4 expression in ccRCC tissue microarrays and survival prognosis. A: Representative images of IHC staining of cancerous tissue and its paracancerous tissue DOCK4 in tissue microarrays of ccRCC patients. Bar = 200 μm。 B: Expression levels of DOCK4 in ccRCC tissue microarrays. C: Survival analysis of patients with high and low expression of DOCK4 in ccRCC tissue microarrays. n = 75, ***P < 0.001, bar = 200 μm.

To assess the clinical significance of DOCK4 up-regulation in ccRCC tissues, we performed immunohistochemistry (IHC) on tissue microarrays from 75 ccRCC patients, and the representative tissue images of ccRCC patients are shown in Figure 2A. Pairwise analysis showed that the mean IHC score for DOCK4 was significantly higher in renal clear cell carcinoma tissues compared with normal tissues (Figure 2B). Among the 75 ccRCC patients, the OS rate of patients with upregulated DOCK4 expression was significantly higher than that of patients with low DOCK4 expression (Figure 2C), which was consistent with the results of TCGA data analysis.

### 3.3. The correlation between DOCK4 expression level and the clinical characteristics of patients with ccRCC

**Figure 3.**
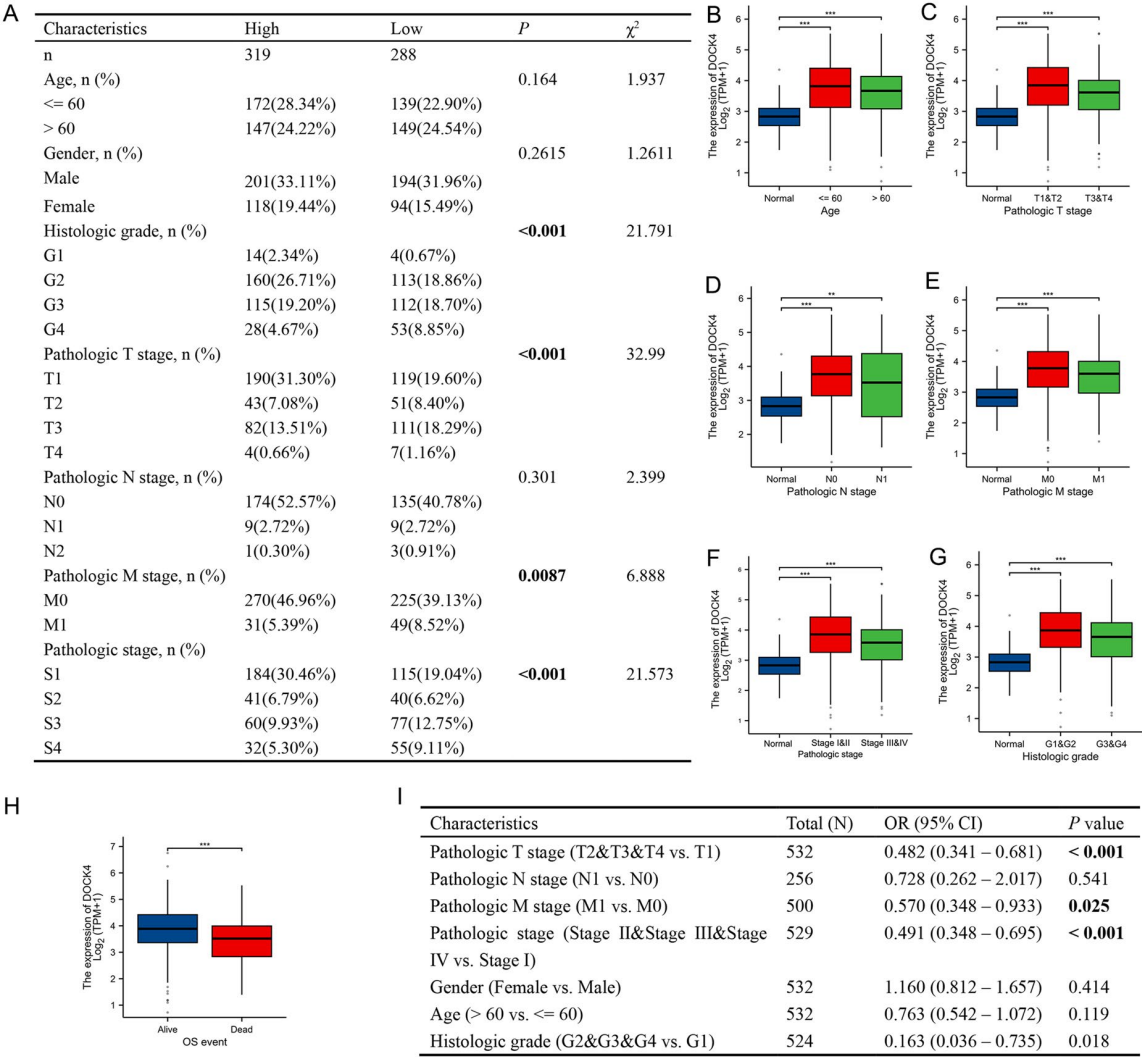
Association of DOCK4 expression levels with clinical characteristics of ccRCC patients. A: Correlation between DOCK4 expression and clinicopathological characteristics of ccRCC patients in the TCGA database and Tissue microarray. B–H: DOCK4 expression in renal clear cell carcinoma was significantly associated with the following characteristics: age (B), ≤60 (n = 264), >60 (n = 268); pathologic T-stage (C), T1&T2 stage (n = 323), T3&T4 stage (n = 206); pathologic N-stage (D), N0 stage (n = 240), N1 stage (n = 16); pathologic M-stage (E), M0 stage (n = 421), M1 (n = 79); pathologic staging (F), stages I&II (n = 323), III&IV (n = 206); histologic grading (G), grades G1&G2 (n = 242), G3&G4 (n = 282); and OS events (H), survival (n = 357), death (n = 175). I: DOCK4 expression correlated with clinicopathological. **P < 0.01, ***P < 0.001

According to the analysis of data in the TCGA database combined with tissue microarray (75 pairs of ccRCC patients), there were differences in clinical stage (T stage) and pathological grade characteristics between DOCK4 high expression group and DOCK4 low expression group (Figure 3A). Interestingly, in ccRCC patients with older age, higher pathological T stage, N stage and M stage, pathological stage and histological grade, the expression level of DOCK4 was lower, but still higher than that in the normal group (Figure 3B–G). Similarly, the expression of DOCK4 in deceased patients was lower than that in surviving patients (Figure 3H), which requires further verification through survival analysis. Furthermore, the logistic regression analysis of the TCGA-ccRCC dataset showed that the expression level of DOCK4 was significantly correlated with pathological T stage, M stage, pathological stage, and histological grade (Figure 3I). Overall, these results suggest that DOCK4 has discriminatory power between tumor and normal samples in ccRCC.

### 3.4. Prognostic value of high DOCK4 in ccRCC

**Figure 4.**
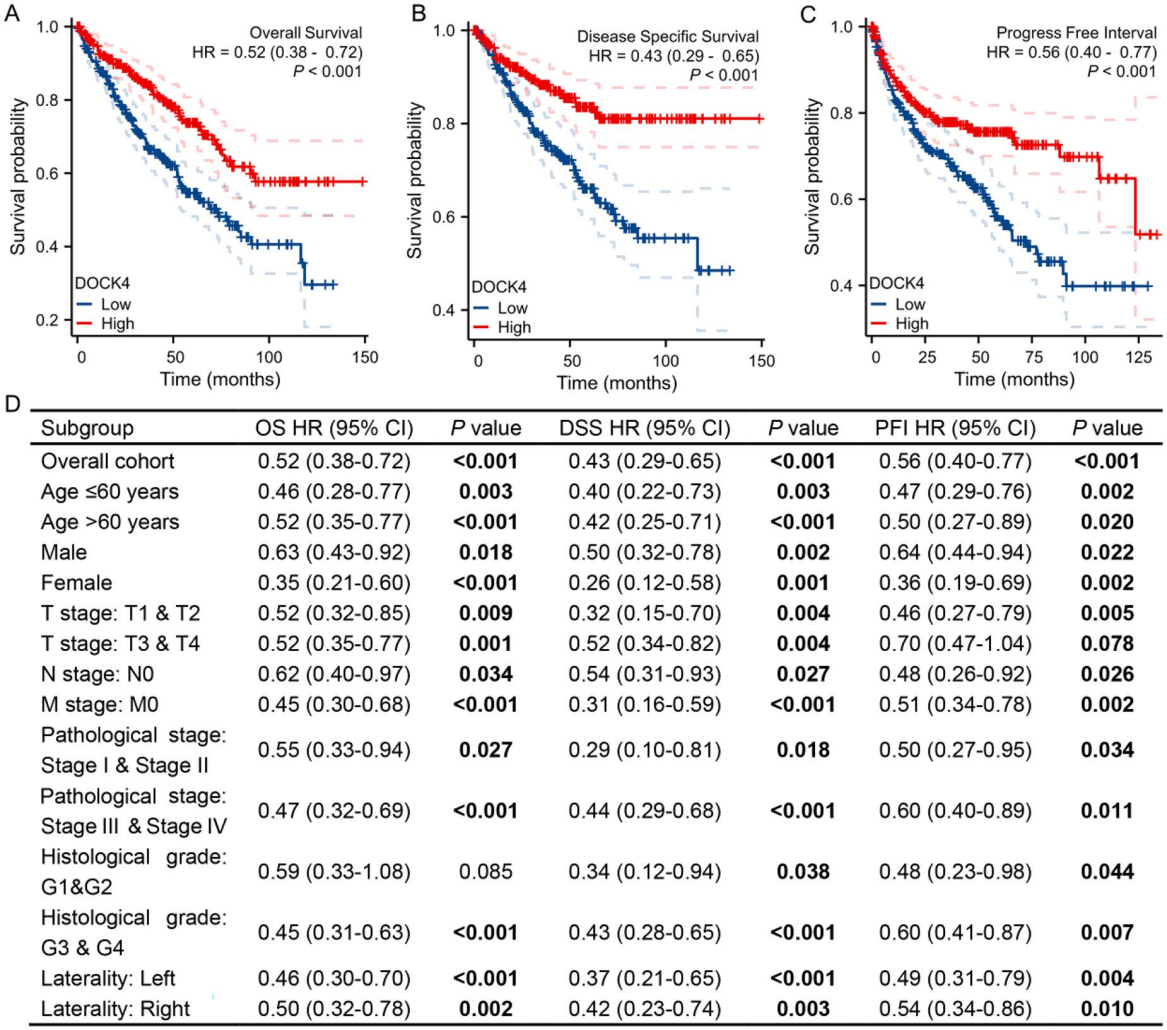
Kaplan–Meier survival analysis to assess the prognostic value of differential DOCK4 expression in ccRCC. A: OS, Overall Survival. B: DSS, Disease Specific Survival. C: PFI, Progress Free Interval. D: OS survival curves of Male; Female; Age: ≤ 60; Age:>60; T1 & T2; T3 & T4; N0; M0; Stage I & Stage II; Stage III & Stage IV; G3 & G4; Laterality (Left); Laterality (Righ). DSS survival curves of Male; Female; Age: ≤ 60; Age: >60; T1 & T2; T3 & T4; N0; M0; Stage I & Stage II; Stage III & Stage IV; G1 & G2; G3 & G4; Laterality (Left); Laterality (Righ). PFI survival curves of Male; Female; Age: ≤ 60; Age: >60; T1 & T2; N0; M0; Stage I & Stage II; Stage III & Stage IV; G1 & G2; G3 & G4; Laterality (Left); Laterality (Right).

To evaluate the prognostic value of DOCK4, the survival curve was plotted using the Kaplan-Meier method. The results showed that the OS, disease-specific survival (DSS) and progression-free interval (PFI) of the DOCK4 high-expression group were significantly better than those of the low-expression group (Figure 4A–C). This study also performed subgroup analyses based on eight clinicopathological characteristics, including age, gender, tumor stage, pathological grading and tumor location. The results showed that in most clinical subgroups, patients with high DOCK4 expression had significantly better OS, DSS and PFI (Figure 4D). These results suggest that high DOCK4 is an effective prognostic factor suggesting a protective role in ccRCC.

### 3.5. DOCK4 as a predictor of survival outcome in ccRCC

**Figure 5.**
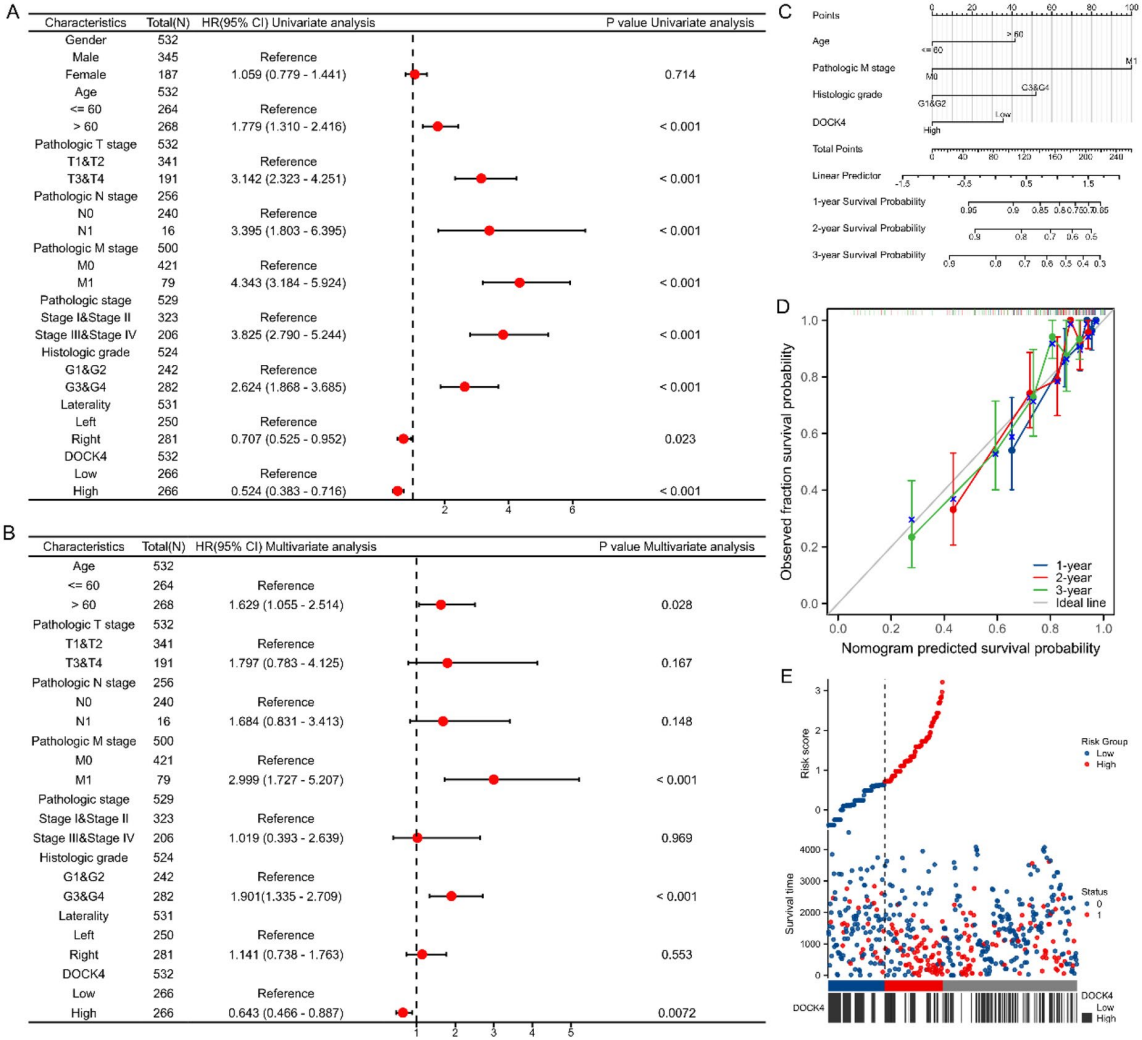
The value of DOCK4 for the prognosis of ccRCC. A-B: Forest plots of OS in ccRCC based on univariate and multivariate Cox regression analysis. C: Construction of prognostic columnar plots. D: Calibration curves of the column-line plots for the prediction of 1-, 2-, and 3-year survival probabilities. E: Risk factor plots showing risk scores and stratification of prognostic models.

The above results indicated that patients with elevated DOCK4 expression showed positive prognosis in ccRCC, prompting the construction of a nomogram integrating clinical characteristics to predict patient survival. One-way and multifactorial Cox regression analyses showed that the age (>60 years), pathological T-stage (T3 & T4), pathological N-stage (N1), pathological M-stage (M1), pathological staging (Stage III & Stage IV), and histological grading (G3 & G4) were the prognostic risk factors; the tumor was located on the right side, and high expression of DOCK4 were prognostic protective factors; independent risk factors were age (>60 years), distant metastasis (M1), histological grading (G3&G4), while DOCK4 high expression was a protective factor (Figure 5A and B). Column line plots constructed based on clinical characteristics and DOCK4 expression levels accurately predicted 1-, 2-, and 3-year survival rates of ccRCC patients (Figure 5C). The calibration curves showed that the predicted values of 1-, 2- and 3-year survival were in close agreement with the actual values (Figure 5D). Risk factor plots showed the correlation between DOCK4 expression and survival outcomes, indicating that higher DOCK4 expression was associated with a lower risk of disease progression and metastasis (Figure 5E).

### 3.6. Functional analysis of DOCK4-related genes highlights its central role in immune regulation

**Figure 6.**
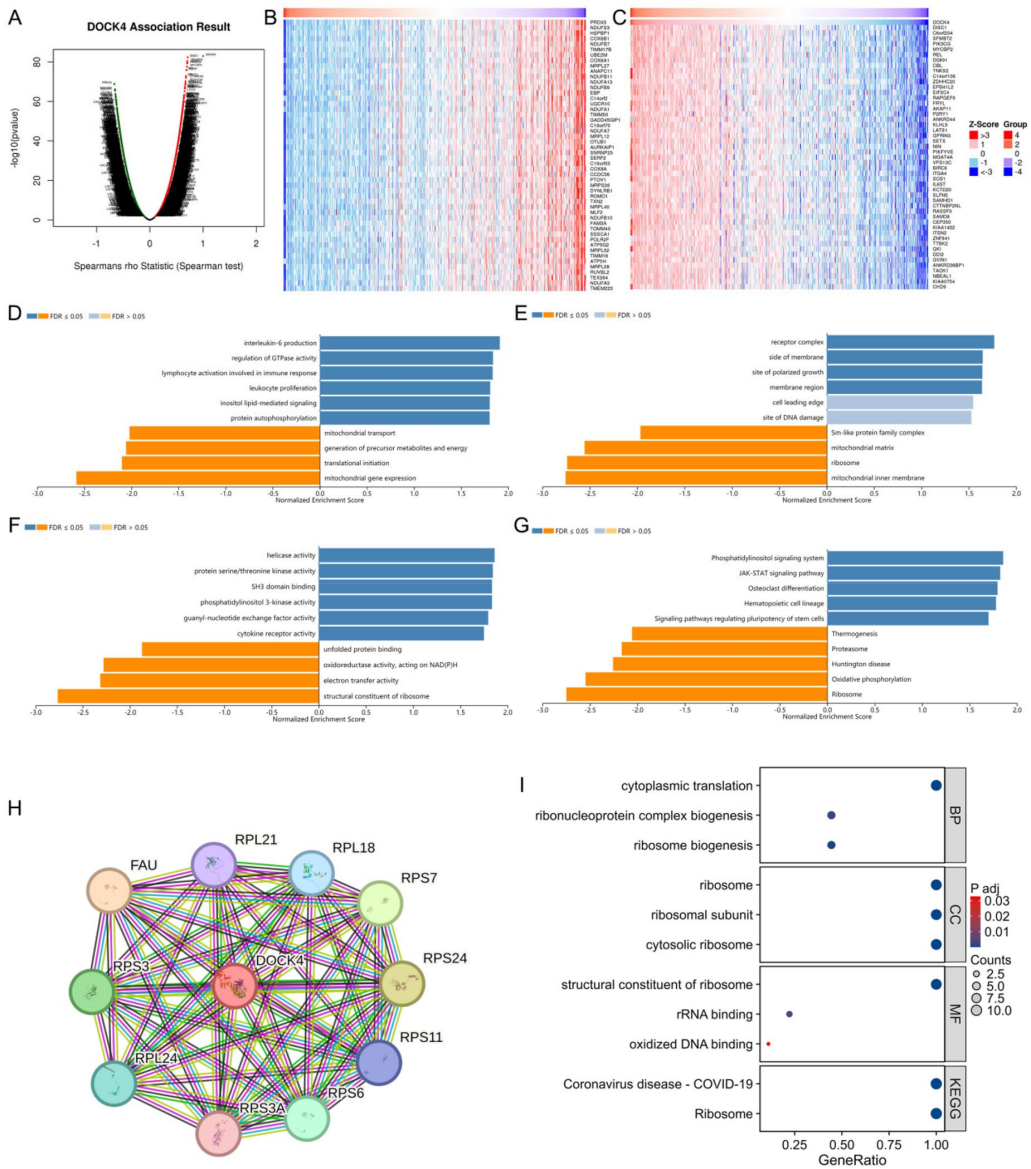
Functional analysis of DOCK4-related genes. A: Genes significantly associated with DOCK4 as screened by Pearson’s test for the ccRCC cohort in the LinkedOmics database. B-C: Heatmap display of the top 50 genes positively and negatively correlated with DOCK4 in ccRCC. Red color indicates positively correlated genes, and blue color indicates negatively correlated genes. D-G: GO annotation of DOCK4 in the KIRC cohort (GO-biological process (D), GO-cellular component (E), GO-molecular function (F) and KEGG pathway (G) analysis. H: Protein-protein interaction networks obtained from the STRING database. I: GO and KEGG enrichment analysis of 10 reciprocal genes of DOCK4.

To elucidate the biological functions of DOCK4 in ccRCC, we performed a comprehensive analysis of its correlated genes using the TCGA-ccRCC cohort. This revealed a vast number of genes significantly associated with DOCK4 expression, including 5,517 positively and 4,184 negatively correlated genes (FDR < 0.001; Figure 6A–C). Additionally, GO analysis highlighted its role in key immune events such as interleukin-6 production (Figure 6D). Beyond immunity, DOCK4 was also connected to fundamental cellular activities including signal transduction (e.g. GTPase regulation), energy metabolism, and ribosomal function (Figures 6D–G). A protein-protein interaction network further identified key partners of DOCK4 (Figure 6H). Collectively, these findings position DOCK4 as a potential regulatory hub in ccRCC, with a prominent role in modulating the tumor immune microenvironment, thereby providing a functional basis for its impact on immunotherapy sensitivity.

### 3.7. Association between DOCK4 expression and immune infiltration in ccRCC

**Figure 7.**
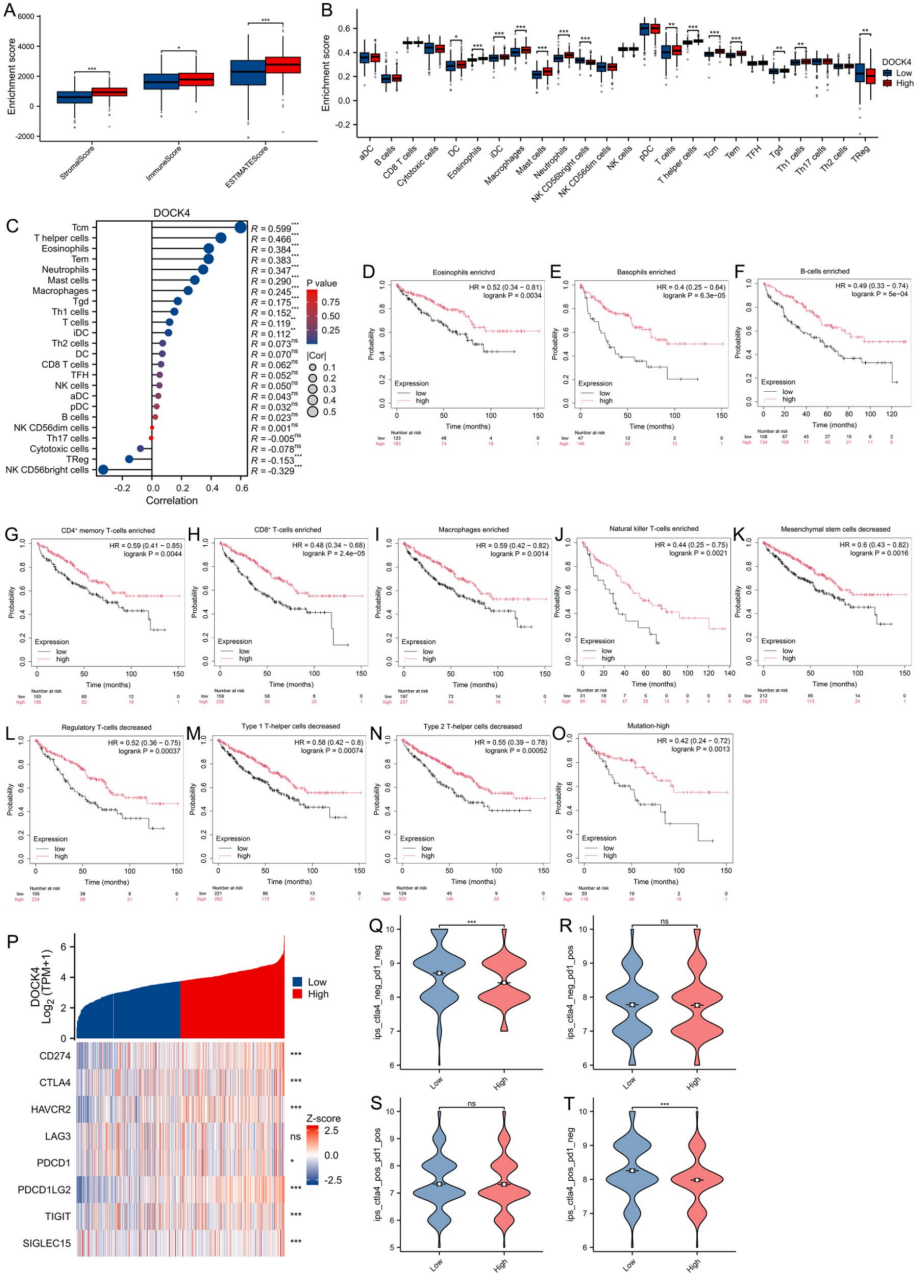
DOCK4 expression correlates with immune infiltration in ccRCC. A: Comparison of stromal scores, immunization scores, and ESTIMATE scores between DOCK4 high-expression and low-expression groups. B: Comparison of immune cell infiltration between DOCK4 high and low expression groups. C: Correlation analysis of DOCK4 expression levels with immune cell infiltration, the size of the points corresponds to the absolute value of Spearman’s rank correlation coefficient. DCs, dendritic cells; aDCs, activated DCs; iDCs, immature DCs; pDCs, plasmacytoid DCs; Th, T helper cells; Th1, type 1 Th cells; Th2, type 2 Th cells; Th17, type 17 Th cells; Treg, regulatory T cells; Tgd, T gamma delta; Tcm, T central memory; Tem, T effector memory; Tfh, T follicular helper; NK, natural killer. D-O: Kaplan-Meier survival curves for comparison of DOCK4 levels in ccRCC based on analysis of various immune cell types. Enrichment group: Eosinophils (D), Basophils (E), B-cells (F), CD4+ memory T-cells (G), CD8+ T-cells (H), Macrophages (I), Natural killer T-cells (J). Decrease group: Mesenchymal stem cells (K), Regulatory T-cells (L), Type 1 T-helper cells (M), Type 2 T-helper cells (N), Mutation-high (O). P: DOCK4 expression and immune checkpoint correlation heatmap. Q-T: Comparison of Immunophenotype Scores (IPS) containing CTLA4-/PD1-, CTLA4-/PD1+, CTLA4+/PD1- and CTLA4+/PD1+ constructed in different DOCK4 expression groups. *P < 0.05, **P < 0.01, ***P < 0.001.

To further explore the effect of DOCK4 on ccRCC progression, we examined its role in the tumor microenvironment. Based on the TCGA database data, we used the ESTIMATE algorithm to calculate the stroma score, immune score, and ultimate score of the tumor samples and assessed the correlation between DOCK4 expression levels and the above scores. As shown in Figure 7A, the stroma score, immune score, and ESTIMATE score of the DOCK4 high-expression group were significantly higher than those of the low-expression group. In the DOCK4 high-expression group, DCs, Eosinophils, iDCs, Macrophages, Mast cells, Neutrophils, T cells, T helper cells, Tcm, Tem, Tgd, and Th1 cells had higher levels of infiltration, and TReg and NK CD56bright cells had lower levels of infiltration (Figure 7B). In addition, we explored the correlation between immune cell infiltration and DOCK4 expression in ccRCC and found that DOCK4 expression was significantly and positively correlated with Tcm, T helper cells, Eosinophils, Tem, and Neutrophils, and infiltration of NK CD56 bright cells was negatively correlated with DOCK4 expression (Figure 7C and Supplemental Figure 1).

Further, we performed a detailed analysis of survival parameters under different immune cell enrichment or reduction conditions. The results showed that among the various immune cell types such as eosinophils, basophils, B cells, CD4+ memory T cells, CD8+ T cells, macrophages, natural killer T cells, MSCs, regulatory T cells, type 1 helper T cells, type 2 helper T cells, and high mutation, a significant difference in survival curves was observed between the high and low expression groups, and the prognosis of high DOCK4 expression was significantly better than that of low expression (Figure 7D–O). These results suggest that the presence or absence of specific immune cells may affect the survival outcomes of groups with different DOCK4 expression levels.

Finally, we performed Spearman’s correlation analysis to explore the association between DOCK4 expression and a variety of immune checkpoints. Notably, the significant correlations observed between DOCK4 and multiple immune checkpoints have drawn our attention (Figure 7P). We investigated the association between DOCK4 and immune checkpoint immunophenotype score (IPS) in ccRCC patients. The analysis showed that the IPS-CTLA4 blocker score was significantly higher in the low-expression group, indicating increased sensitivity to immunotherapy (Figure 7Q–T).

## 4. Discussion

This study presents a comprehensive analysis of the pivotal role of DOCK4 in ccRCC and its clinical significance. By integrating data from TCGA and GEO with tissue microarray validation, we confirmed that DOCK4 exhibited specific high expression in ccRCC tissues (Figure 1). Notably, high DOCK4 expression was linked to more favorable pathological staging (e.g. lower TNM stage, absence of distant metastasis) and significantly prolonged OS, DSS, and PFI (Figures 2–5). This contrasts with the established understanding that the CDM protein family typically promotes metastasis in tumors [25–27]. This paradoxical observation, where a member of a pro-metastatic gene family correlates with a favorable prognosis, suggests that DOCK4 may influence tumor biology through a distinct mechanism in ccRCC. We hypothesize that its function may be tissue-specific or microenvironment-dependent, offering new insights into the exploration of tumor heterogeneity.

This study offers a comprehensive analysis of the functional network of DOCK4-related genes, uncovering its distinct regulatory mechanisms in ccRCC (Figure 6). Unlike classical CDM family proteins like DOCK1 [28], which primarily regulate cell migration, DOCK4 is shown to have a more extensive range of biological functions. GO/KEGG analysis reveals that DOCK4 plays a key role in immune cell regulation and the activation of immune responses (Figure 6D), which might directly correlate with its involvement in remodeling the immune microenvironment (Figure 7). Furthermore, DOCK4 may enhance the secretion of pro-inflammatory factors by activating the JAK-STAT pathway, thereby driving CD8^+^ T cell infiltration, a process that contributes to the survival benefits linked to elevated DOCK4 levels.

The regulation of GTPase activity by DOCK4 (Figure 6D) suggests its role in T cell immune synapse formation, thereby enhancing antitumor immune responses. Notably, DOCK4 is significantly enriched in pathways related to mitochondrial transport (GO-CC, Figure 6E) and oxidative phosphorylation (KEGG, Figure 6G), indicating its potential to reshape the metabolic characteristics of ccRCC. The enhancement of mitochondrial function may counteract the Warburg effect in ccRCC and suppress tumor proliferation. This finding aligns with the favorable prognosis observed with elevated DOCK4 levels. Moreover, the enrichment of ribosome biosynthesis-related genes (STRING analysis, Figure 6H) suggests that DOCK4 preserves cellular homeostasis by inhibiting aberrant protein translation. Additionally, PPI network analysis revealed that DOCK4-interacting genes are significantly enriched in the COVID-19 pathway (Figure 6I), which includes closely overlapping with tumor immune activation. Thus, we hypothesize that DOCK4 can inhibit tumors through immune metabolic regulation.

Through multidimensional survival analysis, this study is the first to confirm that high DOCK4 expression serves as a strong predictor of survival benefit in ccRCC patients. Kaplan-Meier curves clearly demonstrate that the high-expression group significantly outperforms the low-expression group in terms of OS, DSS, and PFI (Figure 4A–C). Subgroup analysis, which included key clinical variables such as age, gender, and tumor stage (Figure 4D), further supports the robustness of its prognostic predictive capability. Even among stage III-IV patients, the high DOCK4 group still demonstrated significant predictive efficacy (*P* < 0.001), highlighting its ability to identify low-risk subgroups within advanced disease. Moreover, DOCK4 acts as an independent prognostic factor, regardless of staging or grading, and when combined with traditional indicators, it significantly enhances predictive accuracy (Figure 5). Patients with high DOCK4 levels may benefit from active surveillance strategies, particularly those with small tumors, thus avoiding overtreatment. In contrast, patients with low DOCK4 levels may require intensified adjuvant therapy, such as postoperative targeted treatments. The marked improvement in DSS (HR = 0.43) directly correlates with better immune therapy response rates. We consider DOCK4 to be a promising candidate for integration into existing immunotherapy prediction systems.

Significant increases in immune cell infiltration (such as DCs, Tem, Th1, etc.) were observed in the DOCK4 high-expression group (Figure 7B), which may enhance chemokine secretion through the immune regulatory axis. Additionally, the reduction in Treg cells (*P* < 0.001) was inversely correlated with NK CD56 bright cells (Figure 7C), suggesting that DOCK4 alleviates immune suppression within the microenvironment. Moreover, the low DOCK4 group demonstrated increased sensitivity to CTLA-4 inhibitors (elevated IPS-CTLA4 score, Figure 7Q–T). This indicates that patients with low DOCK4 expression may benefit from a combined strategy of immune activation and checkpoint inhibition.

Nevertheless, the molecular targets regulated by DOCK4 in immune cell recruitment (e.g. chemokine receptor activation pathways) and its role in immune metabolic reprogramming (e.g. mitochondrial energy metabolism in T cells) remain inadequately understood. Furthermore, predictions of immunotherapy sensitivity based on bioinformatics algorithms (such as the IPS score) have not been validated in cohort studies. Current spatial resolution techniques are limited (e.g. the ESTIMATE algorithm cannot pinpoint immune distribution in tumor subregions), and protein interaction networks (e.g. the ribosomal pathway) lack functional validation. Future research should leverage conditional gene editing animal models, multi-center prospective cohorts, and spatial multi-omics technologies to further investigate these aspects.

In summary, this study highlights the multifaceted biological significance of DOCK4 in ccRCC, demonstrating that its high expression is strongly associated with favorable pathological staging and improved survival outcomes. Additionally, DOCK4 plays a pivotal role in reshaping the tumor microenvironment by regulating immune cell infiltration. We recognize that while our bioinformatic analysis suggests a role for DOCK4 in modulating immune infiltration, this was not validated by multiplex immunohistochemistry in our tissue cohort due to sample limitations. Future studies employing multiplex immunofluorescence (mIF) on a larger cohort are warranted to precisely quantify the spatial relationship between DOCK4 expression and specific immune cell subsets. Functional analysis suggests that DOCK4 is involved in immune response and metabolic regulation pathways, while IPS score data indicate that its expression levels correlate with immunotherapy sensitivity. These findings provide novel insights into the role of DOCK4 in ccRCC. While our findings are indeed promising, the clinical application of DOCK4 is primarily conceived as a prognostic biomarker. This biomarker has the potential to enhance risk stratification and serve as a valuable complement to existing clinical nomograms, rather than functioning as a diagnostic tool.

## Supplementary Material

Supplemental Material

## Data and materials availability statement

All data and materials in the manuscript and supporting information can be made available upon reasonable request from the corresponding author.
