# Supplementary material for: Elevated DOCK4 expression correlates with favorable prognosis and immune infiltration in clear cell renal cell carcinoma
Source: Ann Med. 2026 Mar 13;58(1):2642533. doi: 10.1080/07853890.2026.2642533 (PMC12990261; doi:10.1080/07853890.2026.2642533)
Supplement: Supplemental Material [file IANN_A_2642533_SM9290.docx]

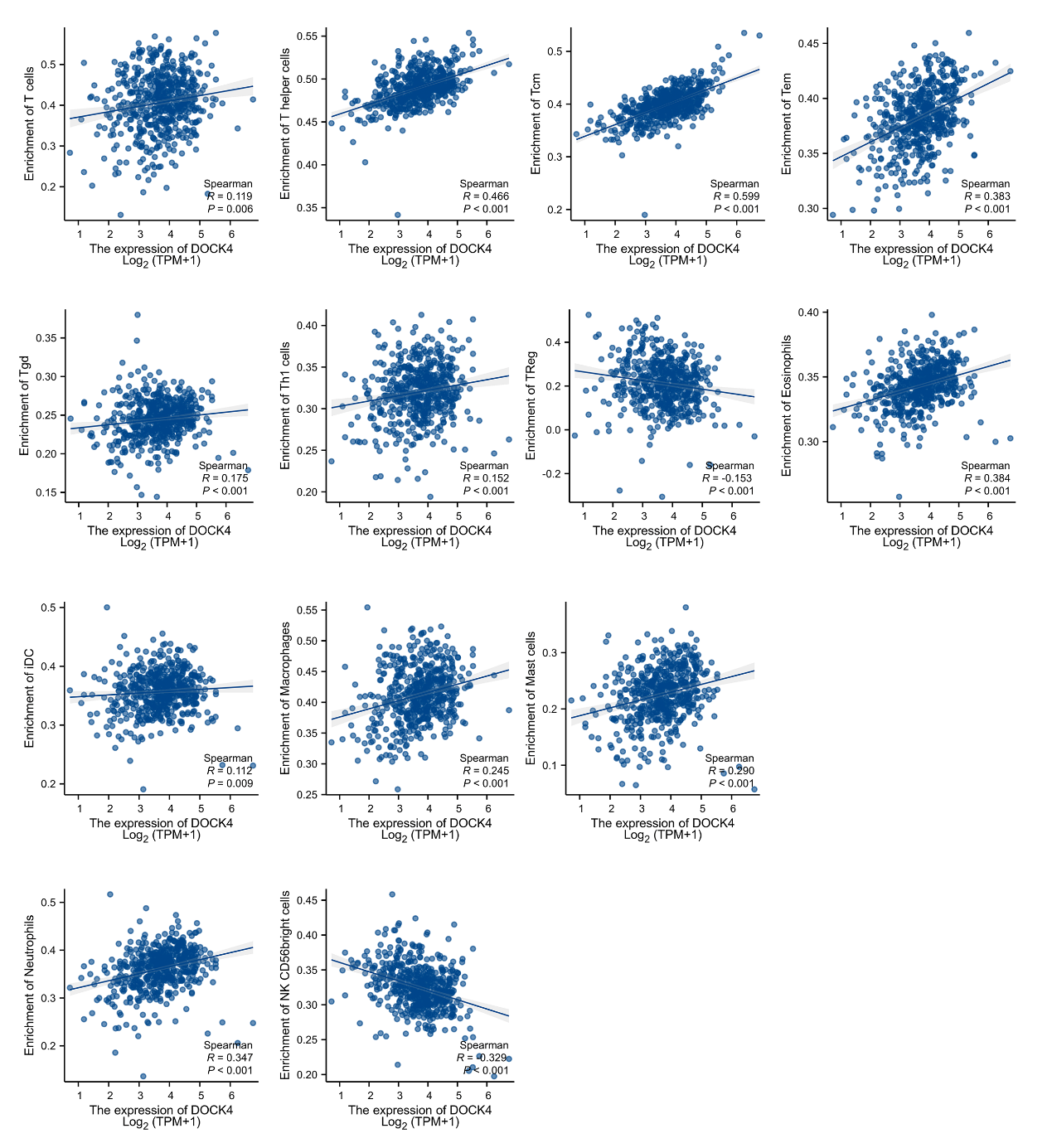
Supplemental Figures

Supplemental Figure1: The correlation analysis between DOCK4 expression levels and immune cell infiltration.

DCs, dendritic cells; aDCs, activated DCs; iDCs, immature DCs; pDCs, plasmacytoid DCs; Th, T helper cells; Th1, type 1 Th cells; Th2, type 2 Th cells; Th17, type 17 Th cells; Treg, regulatory T cells; Tgd, T gamma delta; Tcm, T central memory; Tem, T effector memory; Tfh, T follicular helper; NK, natural killer.
